# Genetic analysis of pericarp pigmentation variation in Corn Belt dent maize

**DOI:** 10.1093/g3journal/jkad256

**Published:** 2023-11-10

**Authors:** Dylan L Schoemaker, Yinjie Qiu, Natalia de Leon, Candice N Hirsch, Shawn M Kaeppler

**Affiliations:** Department of Plant and Agroecosystem Sciences, University of Wisconsin—Madison, Madison, WI 53706, USA; Minnesota Supercomputing Institute, University of Minnesota, Minneapolis, MN 55455, USA; Department of Plant and Agroecosystem Sciences, University of Wisconsin—Madison, Madison, WI 53706, USA; Department of Agronomy and Plant Genetics, University of Minnesota, St. Paul, MN 55108, USA; Department of Plant and Agroecosystem Sciences, University of Wisconsin—Madison, Madison, WI 53706, USA; Wisconsin Crop Innovation Center, University of Wisconsin—Madison, Middleton, WI 53562, USA

**Keywords:** expired plant variety protection, genome-wide association study, hue, maize, quantitative trait locus mapping, pericarp, pigmentation, Plant Genetics and Genomics

## Abstract

The US standard for maize commercially grown for grain specifies that yellow corn can contain at maximum 5% corn of other colors. Inbred parents of commercial hybrids typically have clear pericarp, but transgressive segregants in breeding populations can display variation in pericarp pigmentation. We identified 10 doubled haploid biparental populations segregating for pigmented pericarp and evaluated qualitative genetic models using chi-square tests of observed and expected frequencies. Pigmentation ranged from light to dark brown color, and pigmentation intensity was quantitatively measured across 1,327 inbred lines using hue calculated from RGB pixel values. Genetic mapping was used to identify loci associated with pigmentation intensity. For 9 populations, pigmentation inheritance best fit a hypothesis of a 2- or 3-gene epistatic model. Significant differences in pigment intensity were observed across populations. W606S-derived inbred lines with the darkest pericarp often had clear glumes, suggesting the presence of a novel *P1-rw* allele, a hypothesis supported by a significant quantitative trait locus peak at *P1*. A separate quantitative trait locus region on chromosome 2 between 221.64 and 226.66 Mbp was identified in LH82-derived populations, and the peak near *p1* was absent. A genome-wide association study using 416 inbred lines from the Wisconsin Diversity panel with full genome resequencing revealed 4 significant associations including the region near *P1*. This study supports that pericarp pigmentation among dent maize inbreds can arise by transgressive segregation when pigmentation in the parental generation is absent and is partially explained by functional allelic variation at the *P1* locus.

## Introduction

The US standards for maize grain specify that grade 2 yellow corn will contain a maximum of 5.0% of kernels of other colors (Code of Federal Regulations 2023). Therefore, commercial hybrids grown to produce yellow corn, and their inbred parents, typically have colorless pericarps and yellow endosperm and make up over 98% of the US Corn Belt acreage. Alternatively, white corn or popcorn varieties make up <1% each of the maize acreage in the United States (Darrah et al. 2019). The pericarp of the kernel is maternally inherited, so each kernel of a variety with pigmented pericarp will display the same color. The aleurone and endosperm are zygotic tissues that can also display pigmentation. As those tissues are the result of fertilization, kernel color segregation can be observed in certain types of pollinated ears such as on an ear resulting from a self-pollination of an F_1_ hybrid plant. Pericarp and cob glume pigmentation is most often due to phlobaphenes, while aleurone pigmentation is most often explained by anthocyanin accumulation. However, the inheritance and chemical constitution of the entirety of compounds resulting in the array of possible kernel phenotypes is not fully known. This study focused on pericarp pigmentation in segregating populations from inbred parents including those historically important in US dent maize (*Zea mays* L.) production.

Phenotypic variation for maize pericarp color has long been used to study the fundamental principles of genetic inheritance. Emerson (1917) used pericarp variegation patterns to study inheritance patterns in maize, and Anderson and Emerson (1923) studied the genetic factors underlying red and cherry pericarp. Interestingly, the genes associated with phenotypic variation in pericarp pigmentation commonly are explained by, and correlate with, metabolomic variation (Chatham et al. 2019), suggesting that visible pericarp pigmentation has the potential to be used as a correlated trait when selecting for metabolomic accumulation in maize pericarp tissue. The genes controlling kernel color variation can operate additively, as has previously been described for red pericarp pigmentation in rice where the *Rc* loci on chromosome 7 lead to red pericarp pigmentation and having a functional *Rd* locus on chromosome 1 enhances the intensity of the observed color relative to individuals that also inherit the *Rd* allele (Takahashi 1982; Furukawa et al. 2007). Alternatively, pigmentation can arise via epistatic interaction in a multistep pathway.

One extensively studied pathway in maize is the flavonoid biosynthetic pathway. The pathway leads to the accumulation of anthocyanins, maysin, and phlobaphenes (Morohashi et al. 2012; Sharma et al. 2012). Of those compounds, phlobaphenes are water-insoluble and are responsible for generating a brick red to brown pigmentation in maize pericarps and cob glumes (Grotewold et al. 1991, 1994). The transcription factor *pericarp color1* (*p1*) activates multiple genes involved in the production of phlobaphenes and include *c2* (chalcone synthase), *chi1* (chalcone flavanone isomerase), and *a1* (NADPH dihydroflavonol reductase) (Grotewold et al. 1998).

The *P1* locus has been extensively studied (Styles and Ceska 1975) and encodes an R2R3 Myb-like transcription factor (Grotewold et al. 1994) that generates pigment in cob tissue and pericarp but is dependent upon the allelic status. While over 100 alleles have been identified at the *P1* locus (Brink and Styles 1966; Cocciolone et al. 2001), 4 primary alleles control distinct pericarp and cob glume pigmentation patterns in US maize inbreds: *P1-rr* (red pericarp and red cob glume), *P1-wr*, (clear pericarp and red cob glume) *P1-rw* (red pericarp and clear cob glume), and *p1-ww* (clear pericarp and clear cob glume). Due to the control of a multistep metabolic pathway by *P1*, epistatic segregation is often observed if alleles at this locus are segregating. For example, both the *recessive enhancer of maysin1* (*rem1*) (Byrne et al. 1996, 1998; Lee et al. 1998) and *intensifier 1* (*in1*) gene effect require a functional *P1* allele and exhibit an epistatic interaction with the *P1* locus (McMullen et al. 2001).

In *P1-rr* individuals, pigmentation onset occurs 10–12 days after pollination (DAP) and deeply pigmented pericarps result in an observed phenotype of deep red mature kernels. Pericarp pigmentation in *P1-rw* genotypes is first observed 16 DAP and absent from the glumes. Over the grain filling period, the pericarp of individuals containing either allele will continue to darken such that maximum color intensity is observed at physiological maturity (Zhang and Peterson 2005). The pigmentation pattern is influenced by structural variation at noncoding regions of the *P1* loci (Zhang and Peterson 2006) and the intensity of pigmentation influenced by nongenetic factors such as high light intensity, salinity, or drought stress (Lei et al. 2009). The *P1-rr* allele is a single-copy locus, while the *P1-wr* allele contains multiple tandem repeats with each repeat containing a 6.3 kb genic sequence (Cocciolone et al. 2001) that is heavily methylated relative to the *P1-rr* allele (Chopra et al. 1998). The functional *P1* alleles regulate the flavonoid biosynthetic pathway, a multistep and multienzyme pathway that can result in the accumulation of luteolinidin, phlobaphenes, maysin, or anthocyanins (Sharma et al. 2012).

The *P1-wr* allele is predominant in the US dent germplasm, due to requirements for visibly yellow grain and likely due to potential benefits of an active secondary metabolite pathway in cobs in antibiosis and plant protection. Currently, there is still a limited understanding on the exact frequency of the *P1-wr* allele among dent maize. However, among a collection of lines in the Wisconsin Diversity (WiDiv) panel described by Mazaheri et al. (2019), over 50% of the lines contained clear pericarp and red cob glumes, while <25% contained the canonical *P1-ww* phenotype of clear pericarp and cob glumes. However, there are yellow grain commercial hybrids with a white cob due to the presence of clear cob glumes. In addition, white corn and sweet corn varieties also often have white cobs (*p1-ww* allele) selected due to the undesirable presence of colored flecks of glumes in edible grain and products.

Genetic analysis of pericarp pigmentation is supported by quick and efficient methods to phenotype kernel color. In some cases, pigmentation can be studied as a binary trait based on the presence or absence of color, but in many instances, there is a quantitative range of pigment intensity that must be quantified to accurately describe kernel color variation in maize. The intensity of orange color in maize kernels was phenotyped by visually rating 5,000 recombinant inbred lines (RILs) from light yellow to dark orange and then performing quantitative trait loci (QTL) mapping on the visual ratings (Chandler et al. 2013). Owens et al. (2019) used a colorimeter to quantify variation in orange pigmentation among 1,769 yellow to orange inbred lines in the Ames panel (Romay et al. 2013) and identified 9 loci associated with the carotenoid pathway following a genome-wide association study (GWAS). Alternatively, high-throughput phenotyping methods that capture RGB images can be efficiently utilized to phenotype color variation in plant tissue. Caraza-Harter and Endelman (2020) extracted RGB pixel values from potato (*Solanum tuberosum* L.) images and used the formulas proposed by Smith (1978) to calculate hue, chroma, and light (HCL) as a method to analyze the genetics of potato skin set.

The objective of the current study is to combine genetic analysis with high-throughput phenotyping to study the inheritance pattern and genetic architecture of pericarp pigmentation in dent maize biparental populations formed from elite germplasm. To accomplish this objective, genetic analysis for major gene effects and genetic mapping was used to identify QTL for pericarp pigmentation. We then used the WiDiv panel to identify candidate genes involved in pigmentation and to compare QTL found by parental mapping to potential association regions from a diversity panel.

## Materials and methods

### Plant material

**Fig. 1.**
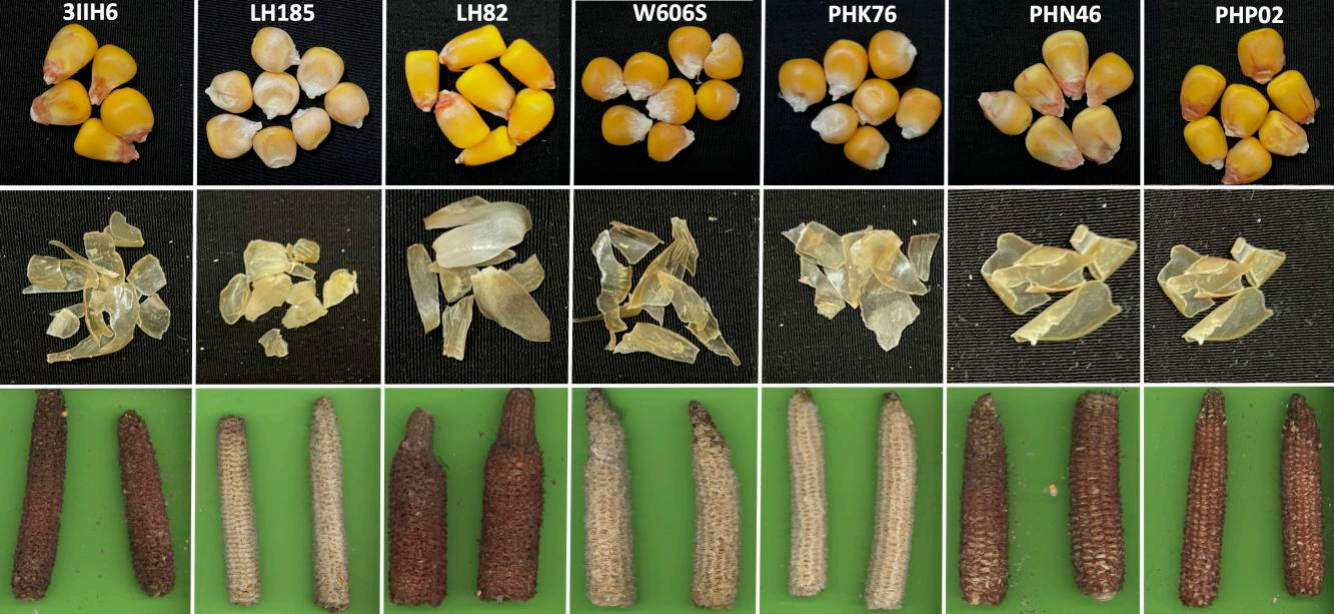
A sample of kernels among the 7 parents used to form the factorial crossing structure that generated the 10 biparental populations used for the analysis. The excised pericarps and cob glume pigmentation of each inbred line are shown below the representative kernels per inbred parent.

A factorial cross of the inbred lines DK3IIH6 (3IIH6), LH185, LH82, and W606S to PHK76, PHN46, and PHP02 (Fig. 1) was formed to generate biparental populations (Table 1). Six of the 7 parents used to produce the populations were derived from expired Plant Variety Protection (ex-PVP) inbred lines. The parents, PHK76, PHN46, and PHP02, were developed by Pioneer Hi-Bred, DK3IIH6 was developed by DeKalb-Pfizer, and LH82 and LH185 are from Holden's Foundation Seed (White et al. 2020). The final parent, W606S, is an inbred line initiated by the Germplasm Enhancement of Maize (GEM) project (Pollak 2003) and released from the University of Wisconsin-Madison Silage Breeding Program. The populations used in this study were produced for other objectives, and they were used in this analysis when we unexpectedly noted pericarp color segregating in the populations. Each of the biparental populations underwent double haploid (DH) induction courtesy of AgReliant Genetics, LLC.

**Table 1.** The name of each biparental population segregating for pigmented pericarp and number of DHs per population shown in parentheses.

	PHK76	PHN46	PHP02
3IIH6	3IIH6 × PHK76	—	—
	(155)		
LH185	LH185 × PHK76	LH185 × PHN46	LH185 × PHP02
	(149)	(153)	(127)
LH82	LH82 × PHK76	LH82 × PHN46	LH82 × PHP02
	(125)	(133)	(119)
W606S	W606S × PHK76	W606S × PHN46	W606S × PHP02
	(122)	(117)	(127)

The populations are formed from the factorial cross of 3IIH6, LH185, LH82, and W606S to PHK76, PHN46, and PHP02. “—” are populations fixed for clear pericarp and were not used for the analysis.

Quantitative variation in pericarp pigmentation was also studied among a sample of 416 inbred lines from the WiDiv panel (Hansey et al. 2011; Mazaheri et al. 2019). The names of the inbred lines from the WiDiv panel used for the analysis are found in Supplementary File S1. The WiDiv panel contained a diverse sample of inbred lines derived from both temperate and tropical environments with 77% of the lines belonging to the dent maize Stiff Stalk, non-Stiff Stalk, or Iodent heterotic groups (Supplementary Fig. 1b). The heterotic group assignments for this germplasm were obtained from Mazaheri et al. (2019).

### Genetic data

Genotyping for the biparental populations was done using genotyping by sequencing (GBS) (Elshire et al. 2011). DNA extraction, GBS library construction, and sequencing were conducted at the University of Wisconsin-Madison Biotechnology Center. DNA from seedlings was extracted using a QIAGEN DNeasy mericon 96 QIAcube HT Kit. Library preparation took place as described by Elshire et al. (2011) with a modification such that 100 ng of DNA was digested using *PstI* and *MspI* (New England Biolabs, Ipswich, MA) instead of *ApeKI*. For Illumina sequencing to be used, barcoded adapters were added via ligation using T4 ligase and PCR was performed to get library concentrations high enough for sequencing. Adapter dimers were removed by SPRI bead purification and sequencing took place using Illumina NovaSeq 6000 and S2 2 × 150 bp flowcells. SNPs were called using the GBSvs2 SNP discovery pipeline in TASSEL 5.0 (Bradbury et al. 2007) and aligned to the B73v5 reference genome (Hufford et al. 2021).

Initial quality control of the GBS data was done using TASSEL 5.0 to filter the original set of 501,243 GBS markers (Bradbury et al. 2007). First, all SNPs with >75% missing data and a minor allele frequency (MAF) <5% were removed. Following, missing SNP markers were imputed using Beagle version 5.1 (beagle.18May20.d20.jar) software (Browning et al. 2018). A numeric matrix consisting of 1's and 0's corresponding to each inbred line having both or no copies of the minor allele was created using the TASSEL plugin -NumericalGenotypePlugin. Next, individuals with >200 crossovers and/or more than 15% missing data were removed from the subsequent analyses using R statistical software (R Core Team 2021). This led to a total of 14,296 genetic markers and 1,327 inbred lines across the 10 biparental populations (Table 1). Using these markers, a genetic distance matrix was calculated using the plugin -DistanceMatrixPlugin in TASSEL 5.0 (Bradbury et al. 2007). Multidimensional scaling was applied to the distance matrix using the function isoMDS() in the R software (R Core Team 2021) package MASS version 7.3–60 (Venables and Ripley 2002) to visualize population structure (Supplementary Fig. 1a).

Genetic markers for the WiDiv panel were obtained from whole genome resequencing described by Qiu et al. (2021). First, the original set of 3,146,253 SNPs were subset to only include 2,511,232 biallelic SNPs. Missing values were imputed using Beagle version 5.1 (beagle.18May20.d20.jar) software (Browning et al. 2018). The 2,511,232 biallelic SNPs from Qiu et al. (2021) were liftover from their B73 reference genome v4 coordinates to the B73 reference genome v5 coordinates such that the SNPs for the WiDiv inbred lines and DHs were aligned to a common reference. The liftover was conducted using CrossMap version 0.6.4 (Zhao et al. 2013), and the B73 reference genome v4 to v5 chain file was downloaded directly from maize GDB (https://download.maizegdb.org/Zm-B73-REFERENCE-NAM-5.0/chain_files/). The uplifting resulted in 2,129,157 SNPs. Additionally, upon initial summary of the resequencing information, we observed that the resequencing genetic data from Qiu et al. (2021) did not contain SNPs at the well-studied genomic region associated with cob glume and pericarp pigmentation in maize, *p1* (Zhang and Peterson 2005, 2006). To ensure SNP information was included within this region, an additional subset of 176 SNPs between 47,922,429 and 48,034,773 bp on chromosome 1 at the *P1* locus were included. The SNPs were obtained from the collection of 46 million high-quality full genome resequencing SNPs described by Grzybowski et al. (2023). The SNPs were downloaded directly from maize GDB (https://www.maizegdb.org/diversity), and all SNPs across the entire *P1* region were subset using variant calling format (VCF) tools version 4.0. Missing values among the 176 genetic markers at *P1* were imputed using Beagle version 5.1 (beagle.18May20.d20.jar) software (Browning et al. 2018). The 2 genetic data sources were merged using the -mergeGenotypeTables plugin in TASSEL. Monomorphic markers and SNPs with a MAF <5% were removed from the merged data set which resulted in a final set of 1,941,521 SNPs for further analysis. The hapmap with the major and minor allele assignments for the inbred lines used for this analysis is found in Supplementary File S2.

### Experimental design

Both, the biparental mapping populations and association panel, were grown in single-row plots. Each row was 3.81 m long by 0.762 m in between rows. DHs from the biparental populations were grown in the field in 2018 at the West Madison Agricultural Research Station in Verona, WI, using a randomized complete block design, blocked by replication. On average, ears were hands harvested from both replications for 33% of the inbred lines. Ears were hand harvested from both replicates for populations crossed to the common parent LH82. For the W606S × PHK76 population and LH185 × PHK76, ears were harvested from both replicates among 22 and 20%, respectively, of the inbred lines. For W606S × PHN46 and W606S × PHP02, 5% of the inbred lines were harvested from both replicates. For the remaining populations, ears were only hand harvested from the first replicate. The association panel was grown at the Arlington Agricultural Research Station in Arlington, WI, in 2018 using a randomized complete block design blocked by replication with 2 replicates per inbred line. Three representative open-pollinated ears were hand harvested from the center of each field plot at physiological maturity. The ears were then dried using forced air until they reached ∼15% moisture content.

### Phenotyping

#### Imaging

The dried ears were shelled, and a 59.15 mL (2 oz) container was filled with maize kernels from the shelled ears. The kernels were evenly spread out on an Epson V700 flatbed scanner for imaging as described by Miller et al. (2017). This process generated an RGB tag image format file (TIFF) for each plot. The shelled cobs were also imaged using the same image analysis procedure as used for the maize kernels. Due to the variation in the size of the maize kernels among inbred lines, each image contained ∼50–80 kernels. The RGB TIFF images were compressed to 10% of the original size and used for subsequent analyses to reduce the computational demand of utilizing the raw images.

#### Observed frequencies and qualitative analysis

**Fig. 2.**
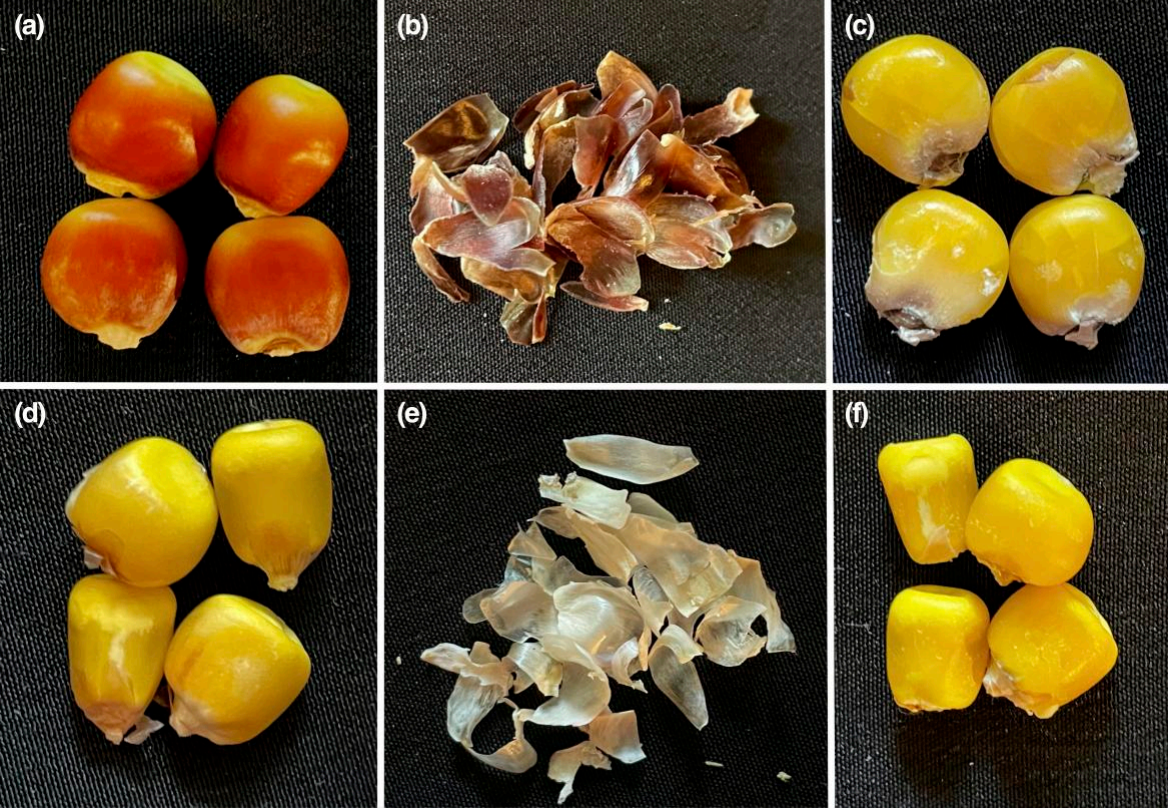
Maize kernels from an inbred line with a) pigmented pericarp and from an inbred line with d) nonpigmented pericarp. b, e) The pericarp tissue is removed from the kernel of both inbred lines. The maize kernels following pericarp excision from the inbred line with c) pigmented pericarp and from the f) inbred line with nonpigmented pericarp.

Pigmentation did not segregate on the ear of the inbred lines so all kernels on the ear displayed pigmentation (Fig. 2a) which is consistent with pigmented pericarp (Fig. 2b) being a maternal tissue (Wright and Neuffer 1989). This allowed us to give each inbred line a binary rating for the presence (1) or absence (0) of pericarp pigmentation by visually inspecting the RGB TIFF files of the maize kernels. Examples of the variation in pigmentation among transgressive segregants are shown in Supplementary Fig. 2. After visual inspection of the maize kernel images, the observed number of inbred lines with pericarp pigmentation was tabulated per population, and the frequency of inbreds displaying pericarp pigmentation per population was calculated by dividing the number of pigmented progenies by the total population size.

A chi-square analysis was conducted to test 6 different genetic models to explain the observed inheritance pattern in pigmentation per population. Genetic models ranged from simple single-gene Mendelian inheritance assuming independent assortment to multigene models including epistasis without linkage (Table 2). All genetic models were tested in R software version 4.1 (R Core Team 2021) using the function chisq.test().

**Table 2.** Expected segregation ratios associated with 6 different genetic models that assumed independent assortment or epistasis without linkage.

Model assumption	Genetic model	Segregation ratio
Independent assortment	Single gene	1:1
	Two additive genes	3:1
Epistasis	Function in 2 genes	1:3
	Function in 3 genes	1:7
	Three genes possible, either a functional gene A or functional genes B and C	5:3
	Three genes possible, a functional gene A is required and either a functional gene B or C	3:5

#### Measuring quantitative variation

Quantitative variation in pigmented pericarp was measured using image analysis and RGB pixel values. To extract the pixel values, RGB TIFF images were initially loaded into R software version 4.1 (R Core Team 2021) with the package magick version 2.7.3 (Ooms 2021) and used to convert each TIFF to a JPEG for use in the package imager version 0.42.10 (Bartheleme et al. 2022). For each JPEG, a binary mask was generated using the “water shed” technique following the workflow described by Bartheleme (2022). The image was first converted to a grayscale with a minimum luminance threshold of 0.4. Thresholding was done using 3,000 subsamples and fitting a linear model that regressed pixel values on both the x and y coordinates of the image to generate a set of predicted values (Bartheleme 2022).

The predicted values were then used as inputs into the package's function threshold() to generate a thresholding value per image. To capture the true shape of each kernel, the functions clean() and fill() were used with pixel values of 1 and 7, respectively, to remove speckles and fill holes in the binary mask and a final thresholding value of 5 was used to remove any debris from the cleaned binary image. The argument values were selected based on arbitrary sampling images, testing multiple input values, and visually inspecting the resulting image segmentation.

**Fig. 3.**
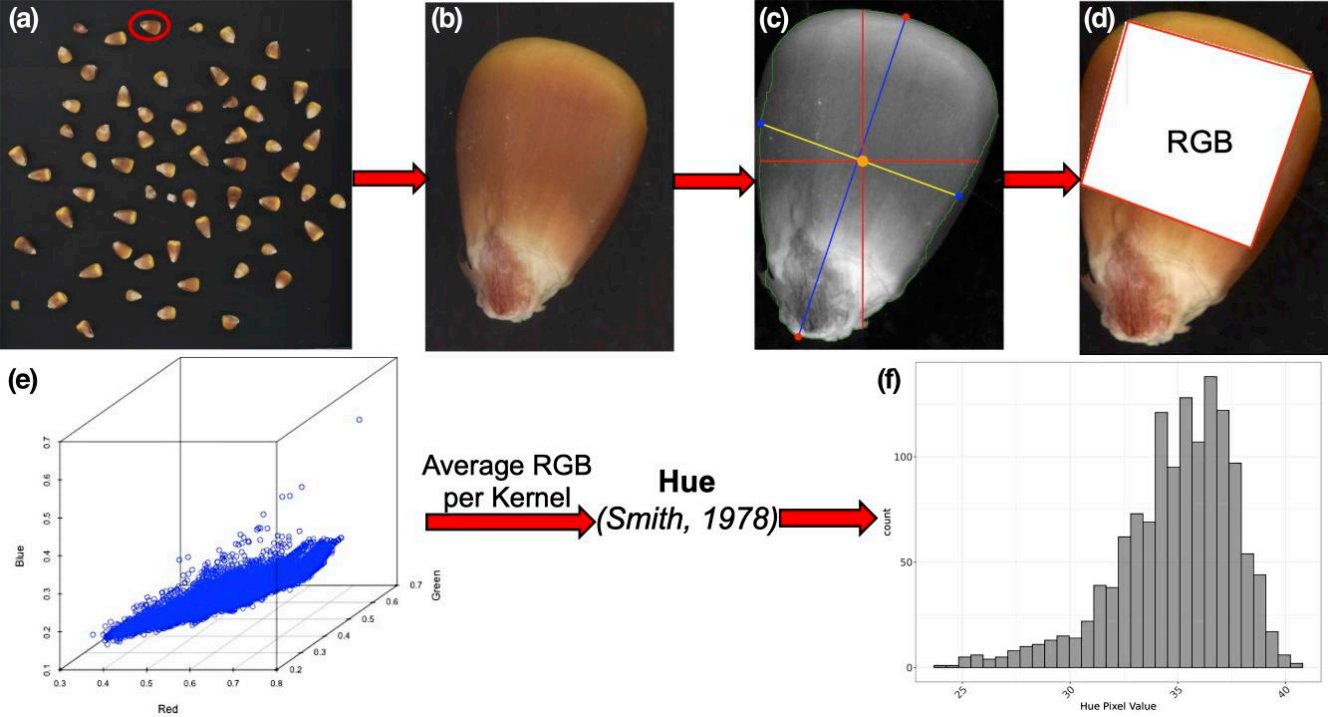
Quantitative variation in pericarp pigmentation was measured using pixel values from a) RGB images of maize kernels. Each b) individual kernel was extracted, and the c) kernel top and based was isolated and used to measure the length and width of the kernel. d) Pixel values from the upper third region of the kernel above the tip and 15% below the base of the kernel were isolated, and the average R, G, and B pixel value among the e) set of pixels within that region was calculated. Hue was then calculated based on the average R, G, and B pixel value for each kernel. The process was repeated for each image and across all sets of germplasm to obtain f) a single hue measurement for each inbred line.

Each of the kernels per image on the black background were isolated and segmented using the function split_connected() (Fig. 3a). Each kernel underwent a series of quality control steps to remove damaged and chipped kernels (Fig. 3b). The function contour() was used to obtain the coordinates of the kernel contour, and kernels with <100 points on the contour were removed as the second quality control step. The center of mass of the kernel was identified from the average x and y value. The tip and base of the kernel were identified by measuring the distance between all pairs of points along the kernel contour, with the longest measurement among any 2 points corresponding to the tip and base of the kernel. The distance between the tip and base of the kernel was used to measure kernel length (Fig. 3c).

The width was identified by making the center of mass a vertex, where the vertex is connected to a second point, the kernel tip. Then, the point on the contour that generated a 90° angle ($\pm 5^{\circ }$) with the center of mass and the tip represented 1 of the 2 width points. A straight line was drawn from the first width point through the center of mass and connected to the adjacent point on the kernel contour to measure the kernel width (Fig. 3c).

Any kernel where the width, major axis, or minor axis could not be obtained were removed from the subsequent analysis. To minimize any color estimation due to the yellow endosperm at the base of the kernel and pigmentation at the tip of the kernel, the average RGB pixel values from the region 15% below the base (cap) of the kernel and 30% above the tip of kernel were segmented (Fig. 3d). Any kernel per image where the length-to-width ratio of the kernel was outside of a 1.5 interquartile range (IQR) was removed. Once the kernels were combined across all inbred lines, a final quality control procedure was applied to remove any kernels where either the R, G, or B color component fell outside of a 1.5% IQR. During quality control, ∼5–35% of the kernels were removed per image, so on average, 50 kernels were remaining per image and used for subsequent analyses.

For phenotypic analysis and genetic mapping, hue was calculated using the average R, G, and B pixel values from the region 15% below the base (cap) of the kernel and 30% above the tip of kernel for each maize kernel based on the formula described by Smith (1978) and calculated using the base R function rgb2hsv() version 3.6.2. The values returned from rgb2hsv() range from (0, 1) so were multiplied by 360° such that the final hue value used for analysis was measured in circular degrees. The values ranged between 0° and 360° (Smith 1978) with smaller hue degrees representing darker color.

The above process was repeated for each inbred line across all available images (Fig. 3f). To confirm that hue calculated from pixel values accurately represented the human perceived color variation observed among transgressive segregants (Supplementary Fig. 2), each image processed through the pipeline was given a 1–5 visual rating for pigmentation intensity where a 1 represented nonpigmented pericarp and a 5 represented dark pigmentation. This rating was conducted by a single scorer to minimize any confounding effects.

### Phenotypic data analysis

Quantitative variation in pericarp pigmentation was analyzed using the linear model in equation one:


(1) $$y_{\mathrm{ij}}=g_{i\;}+r_{j\;}+\varepsilon_{\mathrm{ij}\;},$$

where *y*_ij_ is the phenotypic value for each inbred line, *g*_i_ is a fixed effect for the i^th^ inbred line, and *r*_j_ is a random effect for the j^th^ replication. Best linear unbiased estimators (BLUEs) were calculated with inbred modeled as a fixed effect and replication modeled as a random effect. The residual error represents the genotype-by-replication interaction and was independent and identically distributed with $\;\varepsilon_{\mathrm{ij}}∼N(0,\varepsilon_{\mathrm{ij}})$. The hue BLUEs for each inbred line across the mapping populations and WiDiv panel are provided in Supplementary File S1.

Cullis estimated heritability (Cullis et al. 2006) for hue was estimated using equation 2 across the 10 populations simultaneously and then also estimated for the association panel:


(2) $$h^{2}=1-\frac{\mathrm{PEV}}{2\sigma_{g}^{2}},$$

where PEV is the average standard error of difference between the predicted means obtained from squaring the value returned from avsed using the function predict.asreml() in asReml-R v4 (Butler et al. 2017) and $\sigma_{g}^{2}$ is the inbred component of variance estimated from the data. Significant differences in pericarp pigmentation intensity across the populations were assessed based on a Tukey post hoc test using the Agricola R package version 1.3.5 (de Mendiburu 2019) at a 5% experimental wise error rate.

### QTL mapping

QTL mapping was performed to identify genomic regions associated with quantitative variation in pericarp pigmentation. Genetic mapping was performed in each population independently using the R package R/qtl2 version 0.22.11 as described in Broman et al. (2019). Genotype probabilities were calculated using the function calc_genoprob() through a hidden Markov model. The BLUEs were regressed on the genotype probabilities using the function scan1(), and scan1blup() was used to calculate the random BLUP effects. To declare a QTL as significant, an LOD threshold was established based on 1,000 permutations using the function scan1perm(). The resulting threshold was supplied to the function find_peak() with the drop argument set to 5 to estimate Bayesian credible intervals per QTL peak (Broman et al. 2019). The LOD thresholds per population ranged from 3.07 to 3.44 with the specific thresholds for each population provided in Supplementary Table 1. Supplementary File S3 contains a .R object with genotype probabilities at each genetic marker per population, a combine set of genotype probabilities across all populations, and a physical map. Supplementary File S4 contains an R script as a .Rmd file to conduct QTL mapping per population using all the objects provided in Supplementary File S2 with a detailed description of each object.

### Association analysis

A GWAS was conducted using a subset of inbred lines from the WiDiv panel (Hansey et al. 2011; Mazaheri et al. 2019) to identify significant SNPs associated with quantitative variation in pericarp pigmentation. GWAS was performed using the R package Memory-efficient Visualization-enhance Parallel-accelerated (rMVP) version 1.0.6 as described by Yin et al. (2021). A fixed and random model circulating probability unification (FarmCPU) model was fit to the data that accounted for population structure through both a kinship matrix and principal components. The first 3 principal components were used to control for population structure as the inbred lines primarily grouped into the 3 major dent maize heterotic groups of Stiff Stalk, non-Stiff Stalk, and Iodent (Supplementary Fig. 1b). The principal component analysis was performed using the R package rMVP using the function MVP.Data(). A FarmCPU model was selected as it has higher statistical power for conducting GWAS among germplasm with extensive population (Yin et al. 2021) structure as observed in the WiDiv panel (Hansey et al. 2011; Mazaheri et al. 2019). The maximum number of iterations was set to 10, and the binning method was set to FaST-LMM. All kinship matrices and the principal component analysis were conducted within the rMVP package version 1.0.6 (Yin et al. 2021). During each iteration, the $W^{\prime }W$ matrix was recomputed and the FarmCPU model was fit until convergence occurred (Yin et al. 2021). To declare a SNP as significant while controlling for multiple testing, a Bonferroni correction was applied to maintain a family-wise error rate at 5%. SNPs with a *P-*value <$\frac{0.05}{1,941,521}\;=2.58x10^{-8}$ were declared significant. For each of these significant SNPs, BLUEs were regressed on allele dosage to obtain the percent of phenotypic variation explained by the SNP. For the significant GWAS SNPs, the gene model closest to the SNP was obtained. Linkage disequilibrium (LD) blocks between the proposed candidate gene and significant SNP were then calculated. The square correlation (*r*^2^) for the LD analysis was calculated using the R package gpart version 1.1 with the function LDblockHeatmap() (Kim et al. 2019) using the default arguments. Additionally, a resampling-based GWAS approach was also conducted following. GWAS was performed using a random subset of 80% of the data for a total of 100 iterations. Based on those 100 iterations, the resample model inclusion probability (RMIP) value at each of the significant SNPs was calculated (Valdar et al. 2009).

## Results

### Assessment of qualitative inheritance

Across the 10 biparental populations, 23–57% of the DHs per population were classified as having pigmented pericarp (Table 3). W606S × PHP02 was the only population that best fit a simple single-gene model under Mendelian inheritance as ∼50% of the progeny displayed pigmented pericarp. In the LH185 × PHK76 and W606S × PHK76 populations, 57% of the progeny in each population displayed pigmented pericarp (Table 3). Segregation in these populations best fit a genetic model where either a functional allele at the “A” locus or an epistatic interaction between the “B” and “C” locus lead to pigmented progeny.

**Table 3.** Frequency of pericarp pigmentation and predicted genetic model based on chi-square analysis of observed and expected frequencies for the factorial biparental populations.

Population	Ratio	Pigmented	Clear	Genetic model	*P*-value
3IIH6 × PHK76	0.23	35	120	2 functional genes	0.49
LH185 × PHK76	0.57	85	64	Functional A or functional B and C	0.17
LH185 × PHN46	0.25	39	114	2 functional genes	0.89
LH185 × PHP02	0.32	41	86	Functional A and functional B or C	0.22
LH82 × PHK76	0.40	50	75	Functional A and functional B or C	0.56
LH82 × PHN46	0.28	37	96	2 functional genes	0.45
LH82 × PHP02	0.33	39	80	Functional A and functional B or C	0.29
W606S × PHK76	0.57	70	52	Functional A or functional B and C	0.24
W606S × PHN46	0.32	38	79	Functional A and functional B or C	0.26
W606S × PHP02	0.50	64	63	Single gene	0.93

Segregation in the populations 3IIH6 × PHK76, LH185 × PHN46, and LH82 × PHN46 best fit a 2-gene epistatic model. Under this scenario, both functional alleles at the “A” and “B” loci are required to observe qualitative variation in pericarp pigmentation. Populations W606S × PHN46, LH185 × PHP02, LH82 × PHP02, and LH82 × PHK76 best fit a genetic model where a functional allele at the “A” locus is required and functionality in either the “B” or “C” locus will generate qualitative variation in pigmented pericarp (Table 3).

### Assessment of quantitative variation

Quantitative variation in pericarp pigmentation was studied using hue calculated from RGB pixel values with smaller hue degrees corresponding to darker pigmented pericarp. BLUEs were estimated from the data and were used to study quantitative variation in pericarp pigmentation among the 10 mapping populations and the diversity panel. Across both the mapping populations and WiDiv, the quantitative variable was highly heritable. The range in hue degrees was greater, and the heritability was lower in the WiDiv panel compared with the biparental populations (Table 4). The slightly lower heritability in the WiDiv panel compared with the mapping populations could be due to the greater genetic diversity in the association panel. The quantitative variable was significantly (*P*-value < 0.001) negatively correlated to the 1–5 visual ratings (Table 4).

**Table 4.** Cullis estimated heritability, Spearman rank correlations with visual ratings, and summary statistics for quantitative hue variable across germplasm.

	Heritability	Correlation	Mean	Min.	Max.	Std. error.
Mapping populations	0.93	−0.79	34.88	24.24	40.79	0.07
WiDiv panel	0.89	−0.69	33.96	25.50	41.90	0.15

**Fig. 4.**
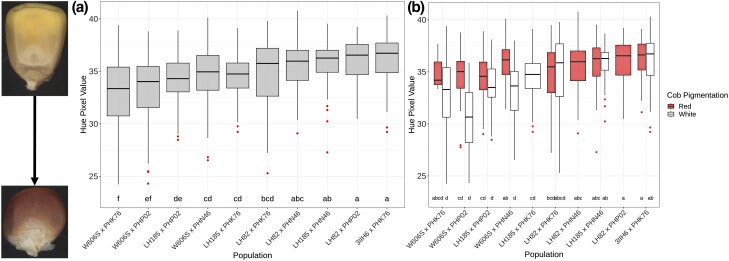
Quantitative variation in pericarp pigmentation between biparental populations measured using RGB pixel values to calculate hue. a) Variation in pericarp hue across 10 biparental population with letters representing significant differences between the average hue value among the 10 populations at a 5% experimental wise error rate following a Tukey post hoc test. b) Variation in pericarp hue across populations according to the cob glume pigment of the progenies per population. Letters represent significant differences between all pairwise combinations of population and cob glume pigment at a 5% experimental wise error rate following a Tukey post hoc test.

Analysis of the quantitative pigmentation variation revealed significant differences in the average hue value across the 10 populations, and W606S populations generally displayed smaller values of hue (Fig. 4a). Based on post hoc analysis, the average hue degrees in W606S × PHK76 was significantly smaller than all LH82-, LH185-, and 3IIH6-derived populations and W606S × PHP02 had significantly smaller (*P*-value < 0.05) average hue degrees than all populations but LH82 × PHK76 (Fig. 4a). However, the 3 W606S-derived half-sib populations were not significantly different from each other. When we subset out just the 462 inbred lines displaying pigmented pericarp, populations with W606S as a parent on average had smaller hue degree.

When averaged across the 3 populations, the hue value of W606S progenies was significantly smaller than the average of LH82, 3IIH6, and LH185 progenies (Supplementary Table 2). Inbred lines that had the darkest pericarp appeared to commonly have no cob glume pigmentation. Interestingly, significant differences in quantitative variation across the populations were only observed among inbred lines that displayed a clear cob glume phenotype, while no differences were observed among lines with pigmented glumes (red cobs) (Fig. 4b). For example, the W606S × PHK76 progenies with clear cob glumes were significantly different than the 3IIH6 × PHK76 progenies with clear cob glumes, but progenies with pigmented cob glumes among the 2 populations were not significantly different on average.

### Genetic mapping

**Fig. 5.**
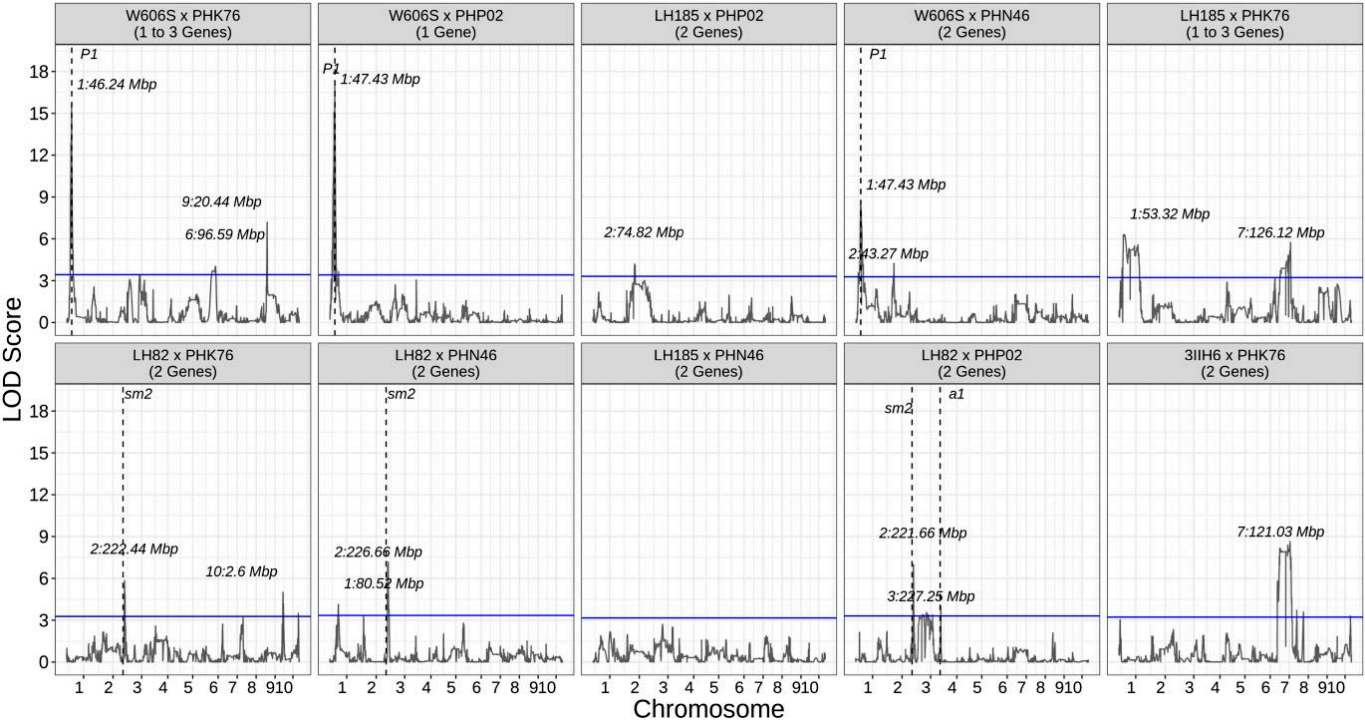
Genetic mapping for quantitative variation in pericarp pigmentation. Quantitative variation was measured based on hue calculated from RGB pixel values, and genetic mapping was conducted independently for each of the 10 biparental populations segregating for pericarp pigmentation. The expected number of functional genes based on the hypothesized qualitative genetic model for each population is shown in parentheses below the name of the population. The blue line shows the population-specific LOD threshold based on 1,000 permutations with the position of significant QTL peaks labeled in megabases (Mbp). The positions of candidate genes within the flavonoid biosynthetic pathway that are near-significant QTL peaks per population are shown with a black dashed line and the name of the candidate gene.

Across the 10 populations, 13 QTL were associated with quantitative variation in pericarp pigmentation and at least 1 significant association was identified in all populations except LH185 × PHN46. The largest QTL peak was observed at 46.24, 47.63, and 47.43 Mbp on chromosome 1 for populations W606S × PHIK76, W606S × PHN46, and W606S × PHP02, 1.50–0.20 Mbp from the *P1* locus (Fig. 5). Analysis of linage disequilibrium decay at the *P1* locus revealed a strong LD block at the gene *Zm00001eb014290* for all 3 W606S-derived populations (Supplementary Fig. 3). For W606S × PHK76, an additional strong association was observed at 20.08 Mbp on chromosome 9. Two additional QTL with LOD scores just exceeding the 1,000 permutation threshold (Supplementary Table 1) were identified on the short arm of chromosomes 6 and 2 for populations W606S × PHK76 and W606S × PHN46, respectively.

**Fig. 6.**
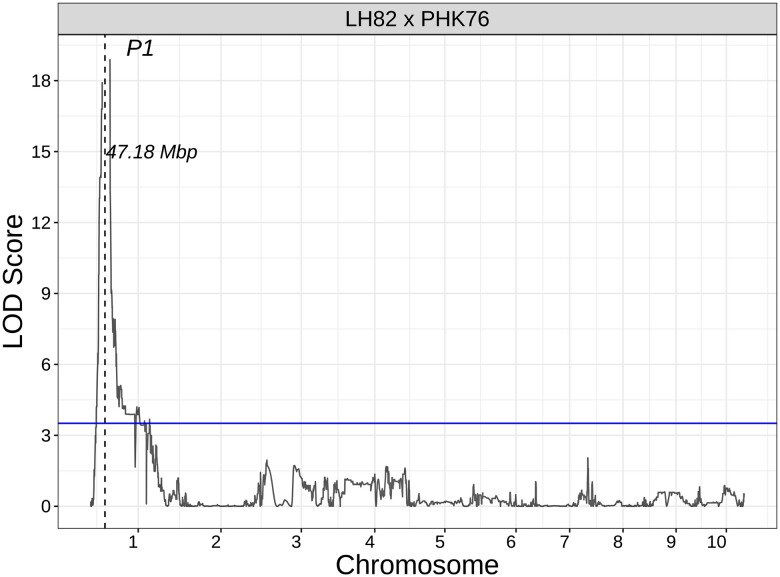
Genetic mapping for the presence or absence (0, 1) of cob glume pigmentation among the LH82 × PHK76 population. The dashed line shows population-specific LOD thresholds based on 1,000 permutations shown as a blue line, and the position of significant QTL peak is labeled in megabases (Mbp). The position of the candidate gene *P1* is shown with a black dashed line.

For populations with LH82 as a common parent, a significant QTL for quantitative variation was observed on the long arm of chromosome 2. The exact position of the QTL changed by population ranging from 221.66 to 226.66 Mbp for LH82 × PHP02 and LH82 × PHN46, respectively (Fig. 5). Among all LH82-derived populations, large LD blocks containing regions in the genome of low recombination were observed near the QTL peaks (Supplementary Fig. 4). LH82 × PHK76 segregates for both pericarp and red cob glume pigment. Within this population, cob glume pigmentation is controlled by a significant QTL peak at the *P1* locus (Fig. 6), and 40% of the 56 inbred lines in the LH82 × PHK76 population with clear cob glumes have pigmented pericarp.

The BLUP effects revealed that the QTL on chromosomes 1 and 2 is inherited from W606S and LH82, respectively, and both are associated with quantitative variation in pericarp pigmentation (Table 5). The average BLUP effects are centered at zero and show deviations from the mean in circular hue degrees. Inheriting the W606S allele on average across the 3 populations led to a 2.11 decrease in circular degrees, while the LH82 effect on average led to 1.08 decrease in circular degrees.

**Table 5.** Estimates of BLUP effects at significant QTL peaks that are associated with quantitative variation in pericarp pigmentation among populations crossed to the common parents W606S and LH82.

Population	Negative allele	Chromosome	Position (Mbp)	Allele effect
W606S × PHK76	W606S	1	46.24	−2.42
W606S × PHN46	W606S	1	47.20	−1.44
W606S × PHP02	W606S	1	47.33	−2.47
LH82 × PHN46	LH82	2	226.66	−1.02
LH82 × PHP02	LH82	2	222.44	−0.88
LH82 × PHK76	LH82	2	222.44	−1.33
W606S × PHK76	PHK76	9	20.44	−1.70

The parental line that contributed the allele associated with pigmented pericarp is listed as the negative allele as the negative allele effect is associated with a decrease in hue.

### Association analysis

For the association analysis, 4 significant SNPs for quantitative variation in pericarp pigmentation were detected and were also identified through the resampling-based GWAS at least 10% of the time (Table 6). The most significant SNP was detected at 47,924,442 bp on chromosome 1 and explained over 7.8% of the phenotypic variation in hue. This SNP had an RMIP value of 38%, the greatest among all the significant associations observed, and the SNP has a MAF of 0.21. The significant SNP was identified within the region of *p1*. The *p1* regions contain 11 tandem repeats and 9 different gene models within the B73 reference genome v5 coordinates. Upon further examination of the repetitive genomic region, the significant SNP was observed closest to the 3 prime untranslated region near the *pericarp color2* (*P2*) locus, a region ∼22.55 kb from the start site of the *P1* candidate gene, Zm00001eb014290. However, this SNP was also in strong LD with the SNP upstream of the *P1* start site near the gene ID Zm00001eb014430, corresponding to the MYB transcription factor 91 (*mybr92*) (Supplementary Fig. 5a). The most significant SNP identified at 47,924,442 is ∼2.0 kb upstream from the *P2* start site which spans from 47,922,429 to 47,930,362 bp.

**Table 6.** Description of significant SNPs associated with quantitative variation in pericarp pigmentation in the WiDiv panel using 1,941,521 whole genome resequencing markers.

Chr.	Pos.	*P*-value	Gene ID	Gene name	MAF	*R* ^2^ *adj*	RMIP (%)
1	47,924,442	1.10 × 10^−16^	Zm00001eb014260	*P2*	0.212	0.078	38
2	149,003,375	1.43 × 10^−13^	Zm00001eb092850		0.272	0.063	23
3	191,736,717	8.56 × 10^−12^	Zm00001eb150000	*ereb77*	0.231	0.074	17
6	164,804,839	1.17 × 10^−11^	Zm00001eb290470		0.091	0.089	21

The significance threshold was set based on a Bonferroni-corrected *P*-value. RMIP values at each significant marker from the full GWAS are shown with the physical position and chromosome number of each association. Gene ID refers to the name of the of candidate gene near the SNP with the canonical gene name provided when available.

Besides the region at *P1*, 3 other significant associations were identified. The SNP at 149,003,375 bp on chromosome 2 had the second largest RMIP value and was observed across 23% of the resamples. Another significant SNP was identified at 191,736,717 bp on chromosome 3 and had an RMIP value of 17%. The SNP was in tight LD with the transcription factor *ereb77*, and the SNP is located near the promoter region of the gene (Supplementary Fig. 5b). The latter single region captured ∼7.5% of the variation in hue. For the remaining significant associations, there were no known candidate genes directly at the significant SNP or within tight LD of the SNP (Table 6). When comparing the significant SNPs identified from the association analysis to the QTLs identified via QTL mapping among the biparental populations, only the association on the short arm of chromosome 1 was significantly associated with quantitative variation in pericarp pigmentation in both sets of materials. In both sources of germplasm, the most significant association was observed near the *P1* region (Supplementary Fig. 6).

## Discussion

Under Federal Regulations for agricultural production in the United States, maize grown for grain that is classified as yellow corn cannot exceed 5% off color. Due to this constraint, continued recurrent selection for grain yield in temperate environments within dent maize germplasm has increased the frequency of the *P1-wr* allele (Frascaroli and Landi 1998), leading to the canonical clear pericarp and pigmented cob glume phenotype. However, we observed a gradient in pericarp pigmentation (Supplementary Fig. 2) among maize inbred lines formed from crosses of selected ex-PVP parents that lacked pericarp pigmentation (Fig. 1). Observing a pigmented phenotype in the progeny and absence of phenotype in the parents is defined as transgressive segregation. To begin understanding the biological basis for transgressive segregation for pericarp pigmentation observed within these 10 populations, we combined qualitative and quantitative genetics to propose and test a set of genetic models to describe the phenotype's inheritance pattern and identified genetic loci associated with variation in pericarp pigmentation.

### Inheritance of pigmented pericarp

According to Mendelian inheritance, if a single gene controlled qualitative variation in pericarp pigmentation, then 50% of the lines in a DH population would display pigmented pericarp (Table 2). However, most of the observed frequencies deviated from this expectation, so 4 of the 6 qualitative models tested described epistatic interactions between alleles at 2 different loci. In 90% of the populations, qualitative variation was best described by a model that assumes 2 or 3 loci are involved with pigmentation, and an interaction between alleles at 2 functional loci leads to pigmented progeny (Table 3). This genetic model has previously been associated with maysin accumulation in maize silks as a functional *P1* locus is required to observe the gene effect for maysin synthesis by *rem1* on chromosome 9 (Byrne et al. 1996, 1998; Lee et al. 1998). *P1* is also epistatic to both *salmon silks1* (*sm1*) and *salmon silks2* (*sm2*) on the long arm of chromosomes 6 and 2, respectively (McMullen et al. 2004; Casas et al. 2016), and *anthocyaninless1* (*a1*) for 3-deoxyanthocyanins accumulation in silk tissue (Grotewold et al. 1994; McMullen et al. 2001; Morohashi et al. 2012). Therefore, we hypothesized that a *P1* regulated epistatic interaction is associated with phenotypic variation in pericarp pigmentation. While the loci *P1*, *a1*, *chi1*, and *c2* are known to be involved in pericarp pigmentation and phlobaphene biosynthesis, many genes are involved in the flavonoid biosynthetic pathway and some of these genes can interact with intersecting pathways (McMullen et al. 2001; Grotewold and Davies 2008). Therefore, genetic mapping was used to determine which loci are contributing to phenotypic variation in pericarp pigmentation.

### QTL mapping for quantitative variation

Based on the qualitative models, we expected that a *P1* regulated epistatic interaction was associated with phenotypic variation in pericarp pigmentation, but variation was only significantly associated with *P1* among W606S-derived populations (Fig. 5). Among the W606S × PHP02 population, pigmentation was expected to be under monogenic inheritance and for the remaining 2 populations, a digenic epistatic interaction was hypothesized. The genetic mapping results supported these qualitative models and demonstrated that both single-gene inheritance of a major functional QTL and digenic epistatic interactions lead to pigmentation variation in the pericarp.

To determine which *P1* allele at the locus was associated with pericarp pigmentation, we compared quantitative variation in pericarp pigmentation among inbred lines with contrasting cob glume pigments (clear or red). We hypothesized that inbreds with pigmented cob glumes would have darker pericarp consistent with presence of a *P1-rr* allele (Zhang and Peterson 2005 and 2006). However, we found that generally inbred lines with clear glumes displayed darker pericarp than individuals with red glumes in populations with W606S as a common parent (Fig. 4b). These results suggest that among these latter populations, the clear cob glume phenotype is associated with darker pericarp. In maize, a pigmented pericarp and clear cob glume phenotype is observed when individuals inherit a *P1-rw* allele. A published *P1-rw* allele arose by recombination between the *P1* locus and its paralogous gene, *P2* (Zhang and Peterson 2005), a locus generally concentrated in the silks (Zhang et al. 2000). However, *P2* can also generate pericarp pigmentation when the *P2* gene promoter is adjacent to the *P1* gene enhancer via transposition of *Ac* and fractured *Ac* (*fAc*) (Sharma et al. 2021). Also, progenies with the darkest pigmented pericarp among W606S-derived populations (Fig. 2) resemble the *P1-rw1077* and *P1-rwCFS342* lines shown in Zhang and Peterson (2006) that are homozygous at the *P1* locus (Brink and Styles 1966; Lechelt et al. 1989; Zhang and Peterson 2005) and pigmentation is concentrated on the side of the kernel while the kernel cap is yellow (Fig. 2a; Brink and Styles 1966). This led to the hypothesis that pericarp pigmentation in W606S-derived populations is associated with the inheritance of a novel *P1-rw* allele.

For the remaining populations, only in the LH82-derived populations was a common QTL region observed among all 3 half-sib populations (Fig. 5). Interestingly, no significant QTL peak was observed near *P1* in these populations, but the qualitative analysis suggested that phenotypic variation in pericarp pigmentation among these populations involves 2 or 3 genes and an epistatic interaction (Table 3). Based on the genetic mapping results among LH82-derived populations, we looked for potential candidate genes within the region between 221.66 and 226.66 Mbp on chromosome 2 (Fig. 5). One gene model within that region is a glycosyltransferase family 61 protein (*Zm00001eb110670*: 221,728,107–221,731,749 bp). A UDP-glycosyltransferase has previously been detected within the *salmon silk2* (*sm2*) (Byrne et al. 1996) mapping interval for silk pigmentation and is expressed higher in *P1-rr* compared with *p1-ww* lines (Casas et al. 2016). *Sm2* would appear as a likely candidate gene as the locus is involved in the flavonoid biosynthetic pathway and regulated by a functional *P1* allele (McMullen et al. 2004), but the locus is ∼13.50 Mbp downstream from the QTL region. To determine if *Zm00001eb110670* was expressed in the pericarp, we examined the gene expression data in the gene atlas developed by Stelpflug et al. (2016) and found that *Zm00001eb110670* is expressed in pericarp tissue at 18 DAP. Further RNA sequencing of pericarp tissue among inbred lines with pigmented and nonpigmented pericarps is needed to identify which of the candidate gene(s) under the QTL peak are associated with pericarp pigmentation.

Two of the 3 LH82 populations are fixed for red cob glume pigmentation, a phenotype regulated by a functional *P1* allele (Grotewold et al. 1991, 1994). The LH82 × PHK76 population segregates for pigmented cob glume pigment, but unlike in the W606S populations, the average intensity of pigmentation in pericarp does not change depending on if the inbred line has clear or red cob glumes (Fig. 4b). The absence of a QTL at *p1* for pericarp pigmentation (Fig. 5) but the presence of a QTL for cob glume pigmentation at the *P1* locus (Fig. 6) suggests that LH82-derived progenies inherited a *P1-wr* allele. Therefore, segregation for pigmented pericarp among LH82-derived populations is not associated with variation at the *p1* locus.

### Comparison of biparental populations and association panel

The qualitative analysis based on chi-squared tests of observed and expected ratios for pigmented pericarp, QTL mapping, and GWAS were 3 complementary approaches to study the genetic architecture of pigmented pericarp among dent maize. The qualitative models and QTL analysis did not always generate consistent results. For example, a 2-gene model was hypothesized for populations LH185 × PHP02 and 3IIH6 × PHK76, but only a single QTL peak was observed (Fig. 5). Our results suggest that complementary approaches to genetic analyses may provide complementary information. Alternatively, the pericarp pigmentation color intensity within these populations varied (Fig. 4), suggesting the phenotype may not necessarily be qualitative and is instead purely a quantitative trait. For example, a QTL peak was detected on the short arm of chromosome 9 for the W606S × PHK76 population and was inherited from the PHK76 parent (Table 6), generating progenies with the darkest pericarp (Fig. 4) that were derived from parents with a nonfunctional *p1* locus (Fig. 1). The QTL peak was in a large LD block (Supplementary Fig. 7) with the candidate gene *deoxy xylulose synthase3* (*dxs3*) (Cordoba et al. 2011). However, fine mapping efforts and expression analysis are now needed to determine the specific gene providing the signal within this QTL region on chromosome 9 among the W606S × PHK76 population. Finally, environmental factors, such as light intensity, can impact pigmentation intensity due to phlobaphene accumulation in the pericarp (Lei et al. 2009) and may have generated discrepancies between the qualitative models and quantitative mapping results.

The differences in the significant associations identified in the WiDiv panel and QTL peaks observed in the biparental populations are most likely due to allelic variation and allele frequency differences between the 2 sources of germplasm. The biparental populations were formed from 6 dent maize–derived lines and 1 dent maize line with 25% tropical alleles (Pollak 2003). Alternatively, 5% of the WiDiv lines were tropical, popcorn, or sweet corn maize inbred lines and 18% of the inbred lines were either developed from public breeding programs or originated outside of the United States (Supplementary Fig. 1b).

Surprisingly, for both the mapping populations and the WiDiv panel, no significant QTL peaks were observed directly at *c2*, *chi1*, or *a1*, potentially due to either a lack of variation at these loci or insufficient statistical power to detect loci with a small effect size. Therefore, our findings suggest that multiple loci contribute to pigmented pericarp variation in dent maize (Supplementary Fig. 6). Similar to the biparental populations, the GWAS results did support that pigmented pericarp in dent maize is partially associated with functional allelic variation at the *P1* region (Table 6). However, structural variation in noncoding regions near *P1* has previously been associated with cob glume variation (Zhang and Peterson 2006) and may be associated with phenotypic variation in pericarp pigmentation. Therefore, structural variants could also be associated with the observed phenotypic variation in pericarp pigmentation. However, further analyses are needed to confirm the role of structural variants and pigmentation inherits among these materials.

### Application in maize breeding

Identifying populations formed from the cross of selected ex-PVP parents segregating for pigmented pericarp offers a unique opportunity for plant breeders to select high-performing maize varieties for economical natural colorants (Chatham et al. 2019). Cultivars with unique hues can serve as a source of natural colorant or be used in value-added food products as color can be extracted from pigmented tissue (Li et al. 2017). Apache Red has been demonstrated as a potential source of germplasm for breeding unique hues into maize varieties for use as natural colorants (Chatham and Juvik 2021), but progenies developed from crosses with exotic germplasm require prebreeding to reduce linkage “drag” from deleterious alleles (Gorjanc et al. 2016). Alternatively, the 10 biparental populations segregating for pigmented pericarp are formed from selected ex-PVPs and an inbred line with ∼25% exotic alleles. Therefore, these populations could be a source of breeding material for developing varieties with dark stable hues as the lines have elite agronomic characteristics and extracting natural colorants from the pericarp captures the secondary metabolites into a single product (Li et al. 2017).

Pigmented maize varieties have potential benefits for both agricultural production and human health. Byrne et al. (1996) reported that a QTL near *P1* for C-glycol flavone content in maize silks can provide resistance against maize earworm (*Helicoverpa zea* Boddie). The accumulation of phenolic compounds, such as flavonoids, has the potential to prevent damage from Fusarium ear rot by increasing the rigidity of the kernel and preventing mycelium from penetrating (Atanasova-Penichon et al. 2016). In an F_3_ maize population, lines homozygous for *P1* had significantly less fumonisin B1 accumulation and higher phenolic acid content compared with lines homozygous for *R1* or *B1/Pl1* (Pilu et al. 2011). These results are supported by Bernardi et al. (2018) who observed that an ancient purple corn variety with a *P1-rr* allele showed significantly greater antioxidant ability and a lower Fusarium infection rate following artificial inoculation. Future research into the relationship between pigmentation variation and metabolite accumulation is warranted to understand any potential agricultural and human health benefits linked to pigmentation within these dent maize mapping populations.

## Conclusions

Combining qualitative and quantitative genetics revealed that epistatic interactions between multiple genetic loci are associated with phenotypic variation in pericarp pigmentation among dent maize–derived populations (Fig. 5). Phenotypic variation in pericarp pigmentation was partially associated with allelic variation at the well-studied locus, *P1*. The locus has been used as a model system to understand the role of cis-regulatory elements in generating phenotypic diversity (Zhang and Peterson 2006) and for studying paramutations (reviewed by Hollick 2017). Our genetic mapping results support that inheritance of a *P1-rw* allele in the progeny is independent of observing the canonical pigmented pericarp and clear cob glume phenotype in the parental generation (Fig. 1). Interestingly, the hypothesized epistatic interactions involving a functional *P1* were not consistently associated with the known genes in the flavonoid biosynthetic pathway *a1*, *chi1*, or *c2* (Grotewold et al. 1998). Therefore, future fine mapping efforts are needed to identify which specific genes are interacting to generate phenotypic variation in pericarp pigmentation among these populations.

## Data Availability

All supplementary materials have been uploaded to Figshare, and RGB images are available upon request. Supplementary Table 1 shows the permutation thresholds used for genetic mapping, and Supplementary Table 2 shows the average hue per common parent. Supplementary Fig. 1 shows the population structure among the mapping populations and WiDiv panel, Supplementary Fig. 2 shows examples of phenotypic variation in pericarp pigmentation among transgressive segregants, Supplementary Fig. 3 shows the LD information among the W606S-derived populations near *P1*, Supplementary Fig. 4 shows the LD information on the long arm of chromosome 2 among the LH82-derived populations, Supplementary Fig. 5 provides the LD information near the candidate genes from the GWAS, Supplementary Fig. 6 shows a Manhattan plot with GWAS results in comparison with significant QTL peaks among the biparental populations, and Supplementary Fig. 7 shows the LD information at the significant QTL peak identified on the short arm of chromosome 9 within the W606S × PHK76 population. Supplementary File S1 contains the hue BLUEs across inbred lines in the mapping populations and WiDiv panel, Supplementary File S2 contains a hapmap file with the inbred lines of interest used for the association analysis in the WiDiv panel, Supplementary File S3 contains a set of R objects for conducting QTL mapping, and Supplementary File S4 contains an HTML file with a description of the objects in Supplementary File S3 and provides the necessary code to conduct each of the steps for genetic mapping. Supplementary material available at Figshare: https://doi.org/10.25387/g3.24446782.
